# Aboveground Feeding by Soybean Aphid, *Aphis glycines*, Affects Soybean Cyst Nematode, *Heterodera glycines*, Reproduction Belowground

**DOI:** 10.1371/journal.pone.0086415

**Published:** 2014-01-22

**Authors:** Michael T. McCarville, David H. Soh, Gregory L. Tylka, Matthew E. O’Neal

**Affiliations:** 1 Department of Entomology, Iowa State University, Ames, Iowa, United States of America; 2 Department of Plant Pathology and Microbiology, Iowa State University, Ames, Iowa, United States of America; INRA, France

## Abstract

*Heterodera glycines* is a cyst nematode that causes significant lost soybean yield in the U.S. Recent studies observed the aphid *Aphis glycines* and *H. glycines* interacting via their shared host, soybean, *Glycine max*. A greenhouse experiment was conducted to discern the effect of *A. glycines* feeding on *H. glycines* reproduction. An *H. glycines*-susceptible cultivar, Kenwood 94, and a resistant cultivar, Dekalb 27–52, were grown in *H. glycines-*infested soil for 30 and 60 d. Ten days after planting, plants were infested with either zero, five, or ten aphids. At 30 and 60 d, the number of *H. glycines* females and cysts (dead females) and the number of eggs within were counted. In general, *H. glycines* were less abundant on the resistant than the susceptible cultivar, and *H. glycines* abundance increased from 30 to 60 d. At 30 d, 33% more *H. glycines* females and eggs were produced on the resistant cultivar in the ten-aphid treatment compared to the zero-aphid treatment. However, at 30 d the susceptible cultivar had 50% fewer *H. glycines* females and eggs when infested with ten aphids. At 60 d, numbers of *H. glycines* females and cysts and numbers of eggs on the resistant cultivar were unaffected by *A. glycines* feeding, while numbers of both were decreased by *A. glycines* on the susceptible cultivar. These results indicate that *A. glycines* feeding improves the quality of soybean as a host for *H. glycines*, but at higher herbivore population densities, this effect is offset by a decrease in resource quantity.

## Introduction

Crop production is at risk for yield loss from both aboveground and belowground herbivores that can occur concurrently and interact through a shared host plant [1], [2]. Recent reviews of aboveground-belowground herbivore interactions have hypothesized that both plant nutrients and common plant defense pathways are important mediators of herbivore interactions [3]–[5]. General hypotheses are proposed including the importance of study location (i.e. field versus greenhouse) [1], feeding guild similarity [3], herbivore arrival time [1], and infestation intensity [3] in determining the outcome of the interaction for each herbivore.

Belowground plant-parasitic nematodes are important yield-reducing pathogens of all major field crops produced in the U.S. [6]. However, their effect on aboveground insects has been sparingly studied, and the reciprocal effect of aboveground insects on nematodes is even less well studied [1]. Johnson *et al.* [1] conducted a meta-analysis that included 123 observations that investigated the interaction between aboveground and belowground herbivores, of which only 11 observations included nematodes. Overall, plant-parasitic nematodes had no observable effect on the performance of aboveground insects, and the reciprocal effect (aboveground herbivores on nematodes) was not examined in the meta-analysis. To what extent the general pattern of above- and belowground herbivores predicts the interaction between nematodes and aboveground herbivores is not known.

Aboveground-belowground herbivore interactions are of particular importance for soybean, *Glycine max* (L.) Merrill, because the crop is challenged by a belowground herbivore, the soybean cyst nematode, *Heterodera glycines* Ichinhoe, and a diverse community of aboveground insect herbivores. *Heterodera glycines* is the leading yield-reducing pathogen of soybean both in the U.S. and worldwide [7], [8]. In the U.S., *H. glycines* is widely distributed throughout all major soybean-producing regions and causes an estimated yield loss of $1.8 billion each year [7]. The population density of *H. glycines* eggs in the soil at the beginning of the growing season is the strongest predictor of yield loss [9]–[12]. Population densities are managed by growing non-hosts (i.e. crop rotation) or *H. glycines*-resistant soybean cultivars. However, crop rotations often consist of two-year rotations, and *H. glycines*-resistant soybean cultivars are mostly derived from a single source of resistance, PI 88788 [13]. Therefore, populations of *H. glycines* persist within the agroecosystem, with infestations of *H. glycines* occuring in 47–83% of fields in the major soybean-producing region of the Midwestern U.S. [14].

Previous research observed that aboveground lepidopteran herbivores were capable of increasing *H. glycines* reproduction on soybean [15], [16]. These studies, however, were conducted using only *H. glycines*-susceptible cultivars and lepidopteran herbivores, which occur rarely as aboveground pests of soybean in the Midwestern U.S. [17]. In this region, the invasive soybean aphid, *Aphis glycines* Matsumura, colonizes fields during early vegetative stages of soybean development with population densities increasing through the reproductive stages of the crop, leading to yield losses of up to 40% in outbreak years [18]. The co-occurrence of *H. glycines* and *A. glycines* is an intriguing system to study as plant-parasitic nematode and aphid infestations both result in changes in induced defense responses and primary plant metabolites [19].

Research exploring the co-occurrence of *A. glycines* and *H. glycines* on soybean suggests that an interaction may occur, but the results of these studies have been incomplete [11], [20]–[22]. *Heterodera glycines* infections are proposed to increase [22], decrease [20], and have no effect on *A. glycines* populations [11], [21]. Furthermore, *H. glycines* infections reduce *A. glycines* alate preference for soybean plants [11], [22]. In addition, *A. glycines* infestations are suggested to both increase [20] and have no effect [21] on *H. glycines* reproduction. Discrepancies in these reports may be due to differences in field versus laboratory settings of the experiments [11], [22], pest population densities, and the inclusion of other pest species in the experimental treatments [20], [21].

McCarville *et al.* [20] and Heeren *et al.* [21] used similar field micro-plots to investigate the effect of *A. glycines* feeding on *H. glycines* reproduction over the course of the entire season. McCarville *et al.* [20] measured *H. glycines* reproduction on soybean infected with either *H. glycines* alone or with *H. glycines, A. glycines,* and *Cadophora gregata* Harrington and McNew, the causal agent of brown stem rot disease. They observed a 500% increase in *H. glycines* reproduction on soybean infested with all three pests. This increase was observed on both *H. glycines*-susceptible cultivars and *H. glycines-*resistant cultivars with the PI 88788 source of resistance. However, McCarville *et al.* [20] did not include a treatment in which plants were exposed to only *H. glycines* and *A. glycines*, and, therefore, could not discern whether the increase in *H. glycines* reproduction was due solely to *A. glycines* feeding.

Heeren *et al.* [21] included treatments in which soybean plants were exposed to *H. glycines* alone or to both *H. glycines* and *A. glycines*, thus making direct observations on the interaction between *A. glycines* and *H. glycines* possible. They did not observe an effect of *A. glycines* feeding on *H. glycines* reproduction. However, their study utilized much lower pest population densities than McCarville *et al.* [20], and in the case of *H. glycines*, densities were often below the limit of detection. Given the discrepancies in pest treatments studied (*A. glycines* alone or in combination with *C. gregata*) and pest population densities utilized between McCarville *et al.* [20] and Heeren *et al.* [21], our goal was to determine whether *A. glycines* feeding by itself could affect *H. glycines* reproduction. In addition we explored whether the population densities of both *A. glycines* and *H. glycines*, which vary widely across the North Central U.S., affect the outcome of the interaction.

## Materials and Methods

In a greenhouse, we manipulated the density of *H. glycines* populations through the use of resistant and susceptible soybean cultivars and *A. glycines* populations through the use of different initial infestation densities. In addition to *H. glycines-*resistant and susceptible cultivars and differential *A. glycines* infestation densities (both described below), pest densities examined were also manipulated by conducting the experiment for different lengths of time. Half of all plants were harvested at 30 d to measure treatment effects on a single generation of *H. glycines* reproduction, and the remaining plants were harvested at 60 d to measure treatment effects after two generations of *H. glycines* reproduction.

For these experiments, a modified version of the Standard Cyst Evaluation-2008 (SCE-08) protocol was utilized [23], in which 125-ml cone-tainers (Stuewe & Sons, Tangent, OR) were arranged in 7.5-l sealed plastic buckets filled with construction sand. The buckets were kept in a water bath to maintain a constant soil temperature between 26.7°C and 28.9°C, which allows for the completion of a single generation of *H. glycines* in approximately 25 d [24].

Cone-tainers were filled with 100 ml of a soil-sand mixture created by adding construction sand to *H. glycines*-infested Eolian Sand type soil. The *H. glycines* population was HG type 0, which is defined by having less than 10% reproduction on all published sources of *H. glycines* resistance (i.e. avirulent to all *H. glycines* resistance genes) [25] and was chosen for its limited ability to reproduce on the PI 88788-derived resistant cultivar utilized in our experiment. Eolian Sand type soil (a fine silt type soil with a high sand content) was used as it consistently permits high *H. glycines* reproduction in the field [26] and is easily washed from soybean roots permitting efficient collection of *H. glycines* females and cysts. The soil was diluted with construction sand to obtain a soil-sand mixture with an approximate population density of 1,000 eggs 100 ml^−1^ of soil. This population density was selected to reduce the likelihood of competition among *H. glycines* females for the nutritional resources of soybean plants. Plants were grown under natural lighting supplemented with 16:8 (L:D) 400 W high-pressure sodium growth lamps and watered as needed.

Two soybean cultivars were used for the experiment, Kenwood 94 and Dekalb 27–52. Kenwood 94 is a *H. glycines*-susceptible cultivar and Dekalb 27–52 is a PI 88788-derived *H. glycines*-resistant cultivar that was used in the field experiment by McCarville *et al.* [20]. In addition to the two soybean cultivars, we used three initial aphid population densities in both the 30 d and 60 d time periods. Aphid treatments were defined by the initial population of *A. glycines* added to each plant (zero aphids, five aphids, and ten aphids). The treatment factors of soybean cultivar and aphid density were fully crossed to create six total treatment combinations per time period. These treatments were arranged in a split-plot design, with the whole plots arranged in a randomized complete block design. It was possible to prevent *A. glycines* from moving between buckets but not between cone-tainers within a bucket, so the whole plot was an individual 7.5-l bucket, with the treatment factor of aphid density assigned to the whole plot. Each bucket contained eight cone-tainers, four per soybean cultivar. Each of the four cone-tainers per soybean cultivar was randomly assigned to one of the two time periods (i.e. 30 d and 60 d). Data were analyzed separately for each time period. Therefore, the split-plot was considered a group of two cone-tainers from the same time point containing the same soybean cultivar, with each individual cone-tainer considered a subsample (two subsamples per split-plot). We conducted three separate runs of the experiment with eight blocks in each of the first two runs, and four blocks in the third. In the third run, all eight cone-tainers in each bucket were allocated to the 30 d group as sufficient statistical power had been achieved in the first two runs of the experiment to test our hypotheses involving the 60-d treatments.

Aphid-density treatments were applied to whole plots when plants reached the first trifoliate or V1 stage [27], which occurred 10 d after planting. Mixed-aged apterous *A. glycines* were transferred from a greenhouse biotype-1 colony (i.e. avirulent to all known *A. glycines* resistance genes, Hill *et al.* [28]) to each plant assigned to the five-aphid and ten-aphid treatments. Each whole plot bucket was then covered with a modified paint strainer (Trimaco, Morrisville, NC) to prevent the movement of aphids among whole plots. *Aphis glycines* populations were then allowed to increase for the remainder of the experiment.

Cone-tainers in the 30-d group were harvested from each whole plot at 30 d after planting, and data were collected as described below. Plants assigned to the 60-d group were transferred with all the soil within their respective 125-ml cone-tainers to 650-ml cone-tainers (Stuewe & Sons, Tangent, OR) after 30 d. The new cone-tainers then were filled to 650 ml with the addition of *H. glycines*-infested soil-sand mixture and placed back into the water bath. These larger cone-tainers prevented soybean roots from becoming tangled and pot bound before the plants were harvested at 60 d, allowing for easier extraction of *H. glycines* females and cysts from roots.

All *A. glycines*, both nymphs and adults, were counted for each plant before the root mass of each plant was soaked in water to dislodge the soil. Roots were sprayed with pressurized water to dislodge *H. glycines* females and cysts, which were captured on a 250-µm-pore sieve positioned below a 850-µm-pore sieve. The total number of females and cysts recovered from each plant was counted under a dissecting microscope. Females and cysts were then ground on a 250-µm-pore sieve using a motorized rubber stopper [29], and released eggs were recovered on a 25-µm-pore sieve nested below a 75-µm-pore sieve. Eggs were suspended in 100 ml of water, and the number of *H. glycines* eggs present in a representative 1-ml sample of solution was counted under a dissecting microscope. The total number of *H. glycines* eggs recovered from each plant was calculated.

### Data analyses

Data collected from the 30-d and 60-d groups of plants were analyzed separately using analysis of variance (ANOVA) with a mixed effects model. The model included the fixed effects of experimental run, block, aphid density, and soybean cultivar. The interactions of run*block, run*aphid density, block*aphid density, and aphid density*soybean cultivar were included as fixed effects. The whole-plot error term of run*block*aphid density was included as a random effect, along with the effect of subsample (i.e. plant nested within aphid density*soybean cultivar). This model allowed us to assess the effects of soybean cultivar, aphid density, and their interaction on the total number of *H. glycines* females and cysts and eggs produced plant^−1^.

The number of *H. glycines* females and cysts plant^−1^ and eggs plant^−1^ were log transformed to meet the assumptions of ANOVA (non-transformed data are presented in all figures). These data were analyzed to determine if soybean cultivar, aphid density, or their interaction affected the number of *H. glycines* females and cysts present or the number of eggs they produced.

Based on the results of our initial analyses, we hypothesized that the effect of *A. glycines* on *H. glycines* reproduction varied with the population density of *H. glycines*. Despite our attempts to limit competition by using an initial low *H. glycines* population density, the numbers of *H. glycines* measured at 30 d and 60 d were high enough to suggest that competition may have occurred among *H. glycines* females. We hypothesized that the competition among *H. glycines* females would be increased with the addition of *A. glycines*. To test this hypothesis, we plotted the effect of *A. glycines* feeding on *H. glycines* population densities across the average *H. glycines* population density in the three aphid treatments. We calculated the effect of *A. glycines* feeding as the percent change in *H. glycines* population densities between the ten-aphid treatment mean and zero-aphid treatment mean for each combination of cultivar and time period (4 total data points). The ten-aphid treatment was selected because it generally represented the strongest effect of *A. glycines* feeding on *H. glycines* reproduction. We plotted the *H. glycines* females and cysts plant^−1^ and eggs plant^−1^ data separately.

## Results

### Aphis glycines populations

Mean *A. glycines* population densities plant^−1^ (± SEM) among the ten-, five-, and zero-aphid density treatments were 278±24, 225±16, and 3±1, respectively for the 30-d group. Upon transfer of the 60-d group plants from the 125-ml cone-tainers to the 650-ml cone-tainers, there were only a few aphids on plants in the zero-aphid treatment. These aphids were removed before the nets were placed back over the buckets. At the conclusion of the 60-d group, *A. glycines* population densities in the ten- and five-aphid treatments had declined to 54±10 aphids plant^−1^ and 99±14 aphids plant^−1^, respectively.

### *Heterodera glycines* population density at 30 d

**Figure 1:**
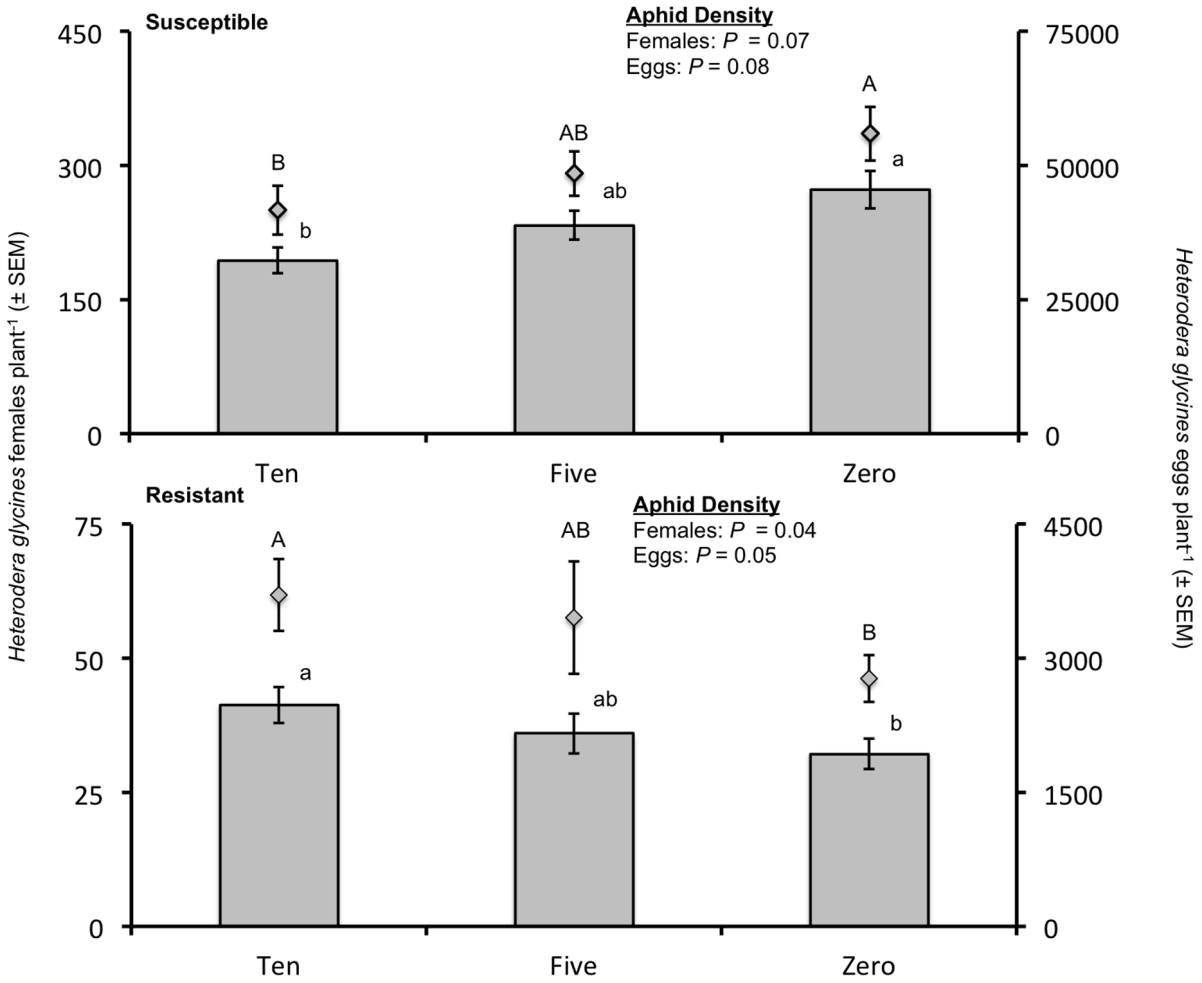
Numbers of *Heterodera glycines* females and eggs recovered plant^−1^ from the *H. glycines*-susceptible soybean cultivar Kenwood 94 and the resistant cultivar Dekalb 27–**52 after 30 d.** Numbers of females are represented by bars with numbers of eggs represented by boxes above the bars. Note the difference in scales used for the two graphs. Three aphid density treatments were established by artificially infesting plants with zero, five, or ten *Aphis glycines* plant^−1^ 10 d after seed was planted. Letters represent significant differences among aphid densities (*P*<0.10), with capital letters assigned to eggs plant^−1^ and lower case letters assigned to females plant^−1^.

Numbers of *H. glycines* females plant^−1^ for the 30-d group varied significantly by experimental run (*F* = 6.27; df = 2,33; *P* = 0.0049), cultivar (*F* = 619.06; df = 1,18; *P* <0.0001), and the interaction of aphid density*cultivar (*F* = 6.18; df = 2,18; *P* = 0.0090). Consequently, the analysis was performed by cultivar to discern the effect of aphid density on the number of *H. glycines* females plant^−1^. On the *H. glycines*-susceptible cultivar, the aphid density treatment factor had a marginally significant effect (*F* = 3.59; df = 2,9; *P* = 0.0715) on numbers of females plant^−1^ with *H. glycines* population densities decreasing with increasing aphid density (Fig. 1). On the resistant cultivar, numbers of females plant^−1^ varied significantly by experimental run (*F* = 11.06; df = 2,11; *P* = 0.0023) and by aphid density (*F* = 4.57; df = 2,9; *P* = 0.0428). The number of *H. glycines* females plant^−1^ increased as aphid density increased on the resistant cultivar, with a 28% increase in numbers of females between the zero-aphid density and ten-aphid density treatments (Fig. 1).

The number of *H. glycines* eggs plant^−1^ for the 30-d group responded similarly to the treatment effects as the number of females plant^−1^. Eggs plant^−1^ varied significantly by experimental run (*F* = 15.55; df = 2,33; *P* <0.0001), cultivar (*F* = 1,129.61; df = 1,18; *P* <0.0001), and the interaction of aphid density*cultivar (*F* = 7.06; df = 2,18; *P* = 0.0055). For the susceptible cultivar, numbers of *H. glycines* eggs plant^−1^ varied significantly by experimental run (*F* = 4.68; df = 2,11; *P* = 0.0338) and the variation in numbers was marginally significant for aphid density (*F* = 3.41; df = 2,9; *P* = 0.0790), with the number of *H. glycines* eggs plant^−1^ decreasing with increasing aphid density (Fig 1). For the resistant cultivar, numbers of eggs plant^−1^ varied by experimental run (*F* = 17.64; df = 2,11; *P* = 0.0004) and by aphid density (*F* = 4.25; df = 2,9; *P* = 0.0502), with the number of *H. glycines* eggs plant^−1^ increasing with increasing aphid density. We observed a 34% increase in eggs on resistant plants initially infested with 10 aphids compared to those assigned to the zero-aphid treatment (Fig. 1).

### *Heterodera glycines* population density at 60 d

**Figure 2:**
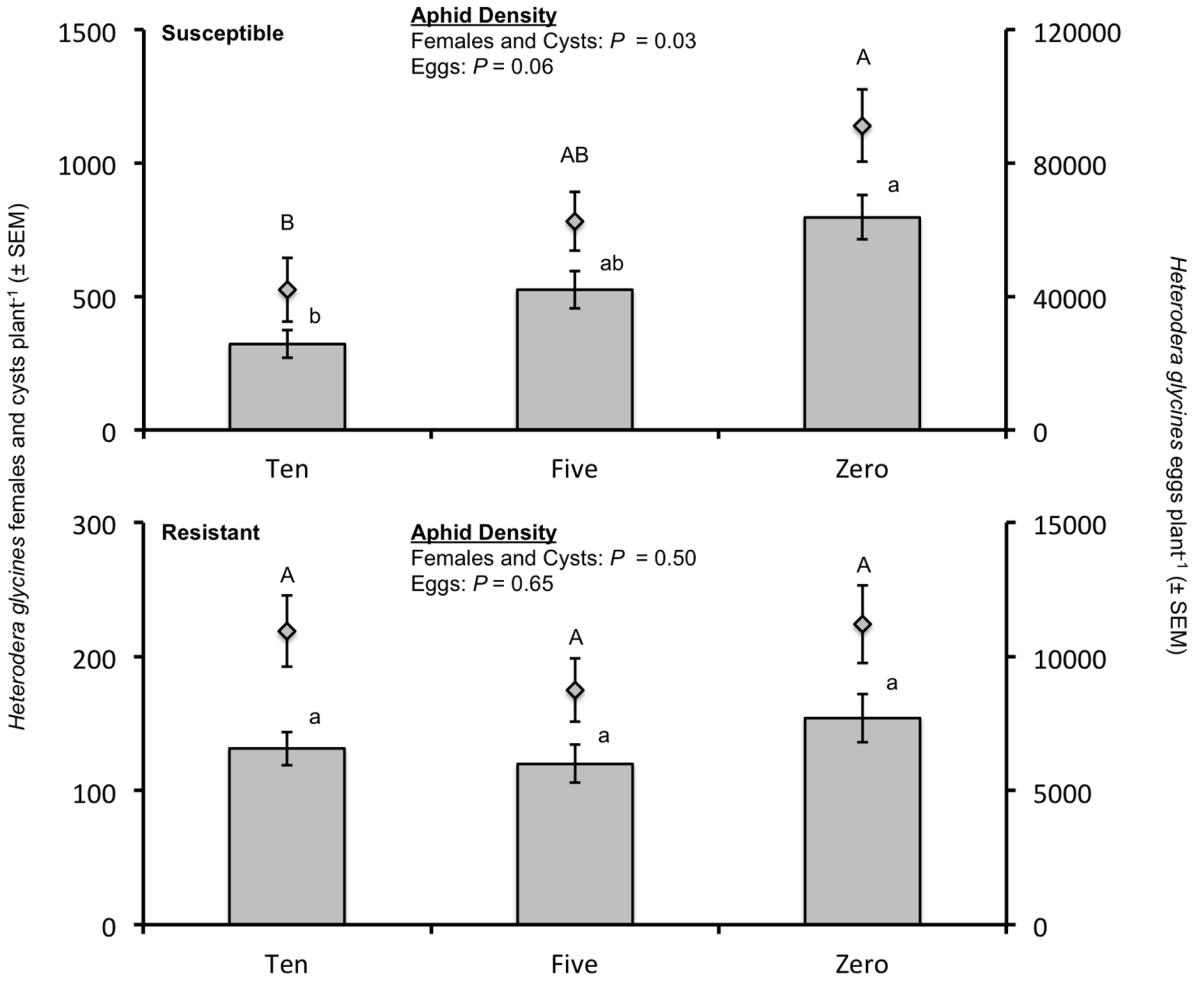
Numbers of *Heterodera glycines* females and cysts and eggs plant^−1^ on the *H. glycines*-susceptible soybean cultivar Kenwood 94 and resistant cultivar Dekalb 27–52 after 60 d. Numbers of females and cysts are represented by bars and numbers of eggs represented by boxes above the bars. Note the different scales used for the two graphs. Three aphid density treatments were established by artificially infesting plants with zero, five, or ten *Aphis glycines* plant^−1^ 10 d after seed was planted. For the susceptible cultivar, aphid density significantly affected the number of *H. glycines* females and cysts plant^−1^ and had a marginally significant effect on numbers of eggs plant^−1^. Letters represent significant differences among aphid densities (*P*<0.10), with capital letters assigned to eggs plant^−1^ and lower case letters assigned to females and cysts plant^−1^.

Numbers of *H. glycines* females and cysts plant^−1^ were affected by cultivar (*F* = 121.76; df = 1,8; *P* <0.0001), and there was a significant aphid density*cultivar interaction (*F* = 4.60; df = 2,8; *P* = 0.0469) for the 60-d group. Consequently, the analysis was performed separately for each cultivar. Aphid density had a significant effect on the numbers of *H. glycines* females and cysts on the susceptible cultivar, with fewer *H. glycines* females and cysts plant^−1^ produced with increasing aphid density (*F* = 5.36; df = 2,8; *P* = 0.0333) (Fig. 2). Aphid density did not affect the number of females and cysts produced on the resistant cultivar (*F* = 0.77; df = 2,8; *P* = 0.4950).

Results from the analysis of *H. glycines* eggs plant^−1^ at 60 d were similar to those obtained from the analysis of females and cysts plant^−1^. The number of eggs plant^−1^ varied significantly by cultivar (*F* = 128.72; df = 1,8; *P*<0.0001), but not significantly by aphid density (*F* = 1.71; df = 2,8; *P* = 0.2414). The interaction of aphid density*cultivar was marginally significant (*F* = 3.56; df = 2,8; *P* = 0.0784). The number of eggs plant^−1^ on the susceptible cultivar varied marginally with aphid density (*F* = 3.94; df = 2,8; *P* = 0.0643), but did not vary by aphid density on the resistant cultivar (*F* = 0.45; df = 2,8; *P* = 0.6521). Overall, the number of eggs plant^−1^ decreased with increasing aphid density on the susceptible cultivar at 60 d (Fig. 2).

### Effect of Heterodera glycines population density

**Figure 3:**
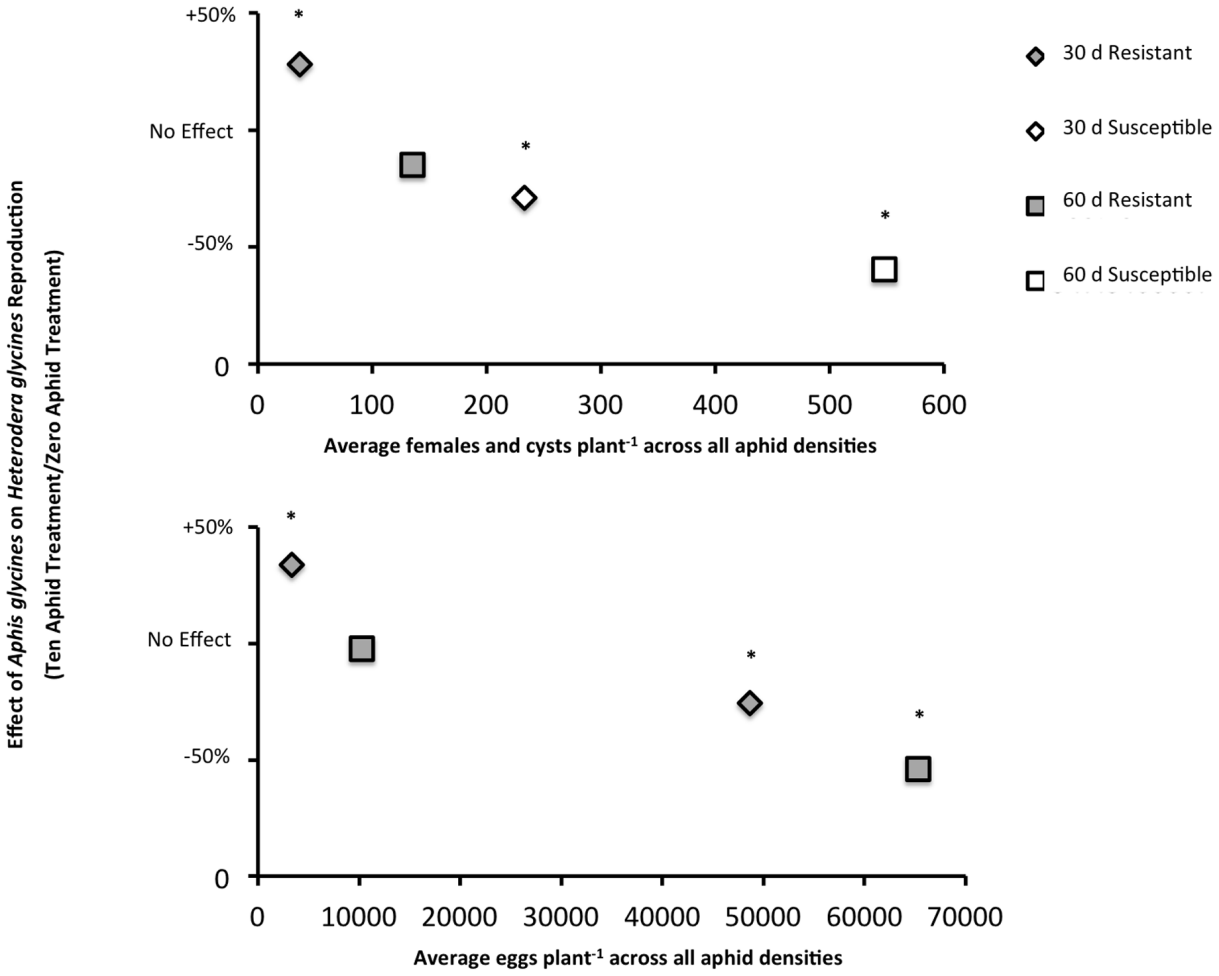
Effect of *Aphis glycines* feeding on numbers of *Heterodera glycines* (a) females and cysts plant^−1^ and (b) eggs plant^−1^ as affected by *H. glycines* population density. The effect of *A. glycines* on *H. glycines* reproduction was calculated as the ratio of the mean of the ten-aphid treatment divided by the zero-aphid treatment. The average number of *H. glycines* females and cysts plant^−1^ and eggs plant^−1^ was calculated as the average of the ten-, five-, and zero-aphid treatment means. *Aphis glycines* increased *H. glycines* reproduction at the lowest *H. glycines* population density, with competition occurring at higher population densities. Asterisks denote data points in which the effect of aphid density was significant at *P* = 0.10 (see figures 1 and 2).

Our data summary analyses of both numbers of *H. glycines* females and cysts plant^−1^ and eggs plant^−1^ revealed that the effect of *A. glycines* feeding on *H. glycines* reproduction was highly dependent on the population density of *H. glycines* (Fig. 3). The trend suggested that as *H. glycines* population densities increased due to either soybean cultivar (susceptible versus resistant) or number of generations (60 d versus 30 d), increasingly negative effects of *A. glycines* feeding on *H. glycines* reproduction were observed. However, at the lowest *H. glycines* population (resistant cultivar at 30 d), *A. glycines* feeding increased *H. glycines* reproduction.

## Discussion

In our experiment, *A. glycines* feeding significantly affected reproduction of *H. glycines.* However, the outcome of this interaction varied significantly with the cultivar and length of experiment. In the 30-d experiment, we observed increased *H. glycines* reproduction on the *H.* glycines-resistant cultivar and decreased reproduction on the susceptible cultivar in response to *A. glycines* feeding. In the 60-d experiment, we again observed decreased *H. glycines* reproduction in response to *A. glycines* feeding on the susceptible cultivar, however we did not observe any effect on the resistant cultivar. We believe the differences in the effect of *A. glycines* feeding on *H. glycines* reproduction to be due to differences in overall pest population densities as mediated by soybean cultivar and experiment length. Support for this conclusion can be found both in the results of the final regression analyses and the *A. glycines* population density data. Our summary analyses (Fig 3.) indicate that higher numbers of *H. glycines* females increased the severity of competition experienced by females upon the addition of *A. glycines* to plants. Competition for limited plant resources may also explain the decline in *A. glycines* population densities from 30 d to 60 d in both the five- and ten-aphid treatments. Soler *et al.* [3] predicted that the population density of herbivores, especially phloem-feeders, a feeding guild that includes aphids and nematodes, would affect the outcome of interactions with other herbivores. More specifically, Soler *et al.* [3] predicted that facilitation would occur at lower herbivore densities and competition at higher densities. The results of this experiment provide evidence supporting this hypothesis. In contrast, Johnson *et al.* [30] found that increasing durations of aphid infestations, and therefore increasing population densities, did not diminish the positive effect of aphid feeding on belowground wireworms. This discrepancy may be a result of the aphid population densities in the Johnson *et al.* [30] experiment not reaching a threshold to induce competition among the wireworms, or it may be due to a difference in how belowground chewing herbivores (i.e. wireworms) and belowground piercing-sucking herbivores (i.e. *H. glycines*) respond to increasing aphid population densities.

Aboveground lepidopteran herbivores are reported to affect belowground plant-parasitic nematodes in soybean [15], [16], [31], with the strength of the effect influenced by insect population density [16], [31]. Generally, these studies reported increasing nematode reproduction for both *H. glycines* and root-knot nematodes (*Meloidogyne* spp.) in response to increasing insect density or damage. This effect is counter to our observation of a variable response of the nematode to increasing aphid population density. This difference in trends may be due to differences in pest population densities in the experiments and the magnitude of their subsequent effect on plant quality, or it may be due to a difference in the resources utilized by the different insect feeding guilds. Both *A. glycines* and *H. glycines* feed from vascular plant tissue, increasing the likelihood for resource competition to occur, whereas lepidopteran herbivores feed on foliage. Therefore *H. glycines* and *A. glycines* could affect each other’s performance both through the removal of shared nutritional resources and activation of related defense pathways [32], [33].

In more recent research, conflicting results concerning the effect of *A. glycines* feeding on the reproduction of *H. glycines* are reported. McCarville *et al.* [20] found that simultaneous infestations of *A. glycines* and the causal agent of brown stem rot disease, *C. gregata*, increased *H. glycines* reproduction. However, Herren *et al.* [21] reported that *H. glycines* reproduction was unaffected by the presence of *A. glycines*. Both of these experiments used small, field micro-plots to measure *H. glycines* reproduction in response to artificial infestations of *A. glycines.* Therefore, it is worth comparing these two experiments to frame the results of our current greenhouse experiment.

McCarville *et al.* [20] observed *H. glycines* reproduction to be 5.24x greater on both *H. glycines-*resistant and susceptible cultivars when plants were also co-infected with *A. glycines* and *C. gregata* compared to plants infected with *H. glycines* alone. This observation was taken from soybean plants infected with *C. gregata* at planting and later infested with *A. glycines* at the early vegetative V3 stage and then comparing end-of-season *H. glycines* egg population densities to beginning-of-season population densities. Therefore, this increase in *H. glycines* reproduction was measured across an entire growing season. In our current experiment, we measured *H. glycines* egg production to be 1.34x greater in the presence of *A. glycines* on the resistant cultivar after 30 d. The 30-d period was a measurement of a single generation of *H. glycines* reproduction. In the U.S., *H. glycines* can complete three to six generations per year [34]. If the 1.34x increase we observed after 30 d occurred across all six generations in the field, we would expect to see a 5.79x increase for the entire year, which is consistent with the findings reported by McCarville *et al.* [20], suggesting that *A. glycines* feeding was primarily or solely responsible for the observed increase in *H. glycines* reproduction in that field micro-plot experiment. It is also noteworthy that, although *H. glycines* resistant and susceptible cultivars supported significantly different *H. glycines* populations in McCarville *et al.* [20], these populations responded similarly to *A. glycines* feeding (i.e. *H. glycines* population densities increased). Therefore, *A. glycines-*mediated competition for resources with *H. glycines* may not occur in the field due to the lower *H. glycines* population densities present in field environments. Supporting this conclusion are the *H. glycines* egg population densities we observed in the current experiment, 55,941 and 91,209 eggs 100cc soil^−1^ in the 30-d and 60-d SCN-susceptible cultivar treatments, respectively, and the average end-of-season *H. glycines* egg population densities in Iowa soybean fields, 2,438 eggs 100cc soil^−1^ (maximum 34,975 eggs 100cc soil^−1^) [13], [35]–[41]). This conclusion is consistent with the findings of Johnson *et al.* [1], specifically that negative effects of aboveground herbivores on belowground herbivores are more likely to be observed in laboratory studies than field studies.

Heeren *et al.* [21] manipulated the population densities of both *H. glycines* and *A. glycines* using a full factorial treatment arrangement of resistant and susceptible lines (i.e. susceptible to both, resistant to both, resistant to *A. glycines*, and resistant to *H. glycines*). They did not detect an effect of *A. glycines* feeding on *H. glycines* reproduction on any of the soybean lines. This result may be due, at least in part, to the extremely low pest population densities present in their study, including <100 *H. glycines* eggs 100cc soil^−1^ and <100 cumulative aphid days (i.e. <10 aphids plant^−1^ for <10 d) for some soybean lines.

Given the results of our current experiment and the previous results of McCarville *et al.* [20] and Heeren *et al.* [21], we propose the following model to explain the effect of *A. glycines* on *H. glycines* reproduction. *Aphis glycines* feeding increases the quality of soybean as a host for *H. glycines* through the manipulation of plant defenses [33] and/or a change in nutrient content [42]. An estimated 28–56% of *H. glycines* juveniles that penetrate susceptible plants reach adulthood [43], [44]. We propose that *A. glycines* feeding increases the percentage reaching adulthood irrespective of the cultivar’s resistance to *H. glycines*. At the 30-d time point in our experiments, *H. glycines* females which reached adulthood would have established their feeding site before aphids were added to plants. Therefore, *A. glycines* did not affect juvenile *H. glycines* penetration or feeding site establishment. *Aphis glycines* increased numbers of *H. glycines* females and eggs, but had no effect on fecundity or eggs female^−1^ (analysis not shown). Therefore, the effect of increased *H. glycines* reproduction observed in our experiment was likely due to an increased number of females. This increase could be through an increased ability of the nematodes to obtain nutrients from the feeding site (i.e. change in nutrient content) or to sustain the feeding site (i.e. change in plant defenses). If an increase in numbers of *H. glycines* females is due to a change in plant defense, this is likely due to a suppression by *A. glycines* of a broad-based, general plant defense to nematodes that is not mediated by *rhg* genes. This interaction, however, is density dependent, with *A. glycines* increasing *H. glycines* reproduction at all pest densities except at very low *A. glycines* population densities (<10 aphids plant^−1^), where aphid feeding has no effect on *H. glycines* reproduction (see [21]), or at high pest population densities (see Fig. 3), where *A. glycines* and *H. glycines* compete for limited nutritional resources.

Going forward, it will be essential to determine under what range of field conditions *A. glycines* feeding leads to an increase in *H. glycines* reproduction or competition with *H. glycines*. It is also necessary to determine whether abiotic factors, such as drought, soil pH, or soil nutrient content can affect the outcome of the interaction indirectly by mediating host plant quality. Finally, given the widespread distribution of both *A. glycines* and *H. glycines* and the economic significance of both pests, it will be important to explore the need for an integrated management approach that mitigates yield reductions that occur both from *A. glycines* removing plant nutrients and from increasing the population density of *H. glycines*. Therefore, a multi-location field study is warranted to investigate this potentially significant aboveground-belowground interaction across a diversity of aphid population densities and infestation timings, nematode population densities, and abiotic conditions.
